# Arising of autoimmune gastritis after *helicobacter pylori* eradication in an elderly female patient

**DOI:** 10.1093/omcr/omae178

**Published:** 2025-01-18

**Authors:** Takayoshi Kiba, Soichiro Nose

**Affiliations:** Department of Internal Medicine, Saiseikai Kibi Hospital, 584-1 Takamatsuharakosai, Kita-ku, Okayama-shi, Okayama 701-1334, Japan; Department of Life Sciences, Faculty of Science, Okayama University of Science, 1-1 Ridai-Cho, Kita-Ku, Okayama-shi, Okayama 700-0005, Japan; Department of Pathology, Okayama Saiseikai General Hospital, 1-17-18 Ifuku-Cho, Kita-Ku, Okayama-shi, Okayama 700-8551, Japan

**Keywords:** autoimmune gastritis, biopsy, eradication, infiltrating cells, *helicobacter pylori*

## Abstract

Autoimmune gastritis (AIG) is a chronic condition in which the body’s immune system mistakenly attacks the stomach lining, specifically targeting parietal cells that produce stomach acid and intrinsic factors. After the *H. pylori* infection was eradicated, AIG developed in an elderly woman with symptoms of the disease. 1.5 years after eradication, esophagogastroduodenoscopy revealed remnants of the oxyntic mucosa sticky adherent dense mucus and scattered minute whitish protrusions at the greater curvature of the gastric corpus. Biopsy specimens from the greater curvature site of the gastric corpus before *H. pylori* eradication revealed neutrophilic cells in the superficial mucosa of the stomach that were mildly inflammatory and infiltrating. With the removal of *H. pylori*, the number of infiltrating inflammatory neutrophilic cells in the superficial mucosa decreased, whereas that of infiltrating lymphocytes increased in the sub-superficial mucosa. This case suggests that further studies regarding the detailed time course of AIG are required.

## Introduction

When autoimmune gastritis (AIG) coexists with *H. pylori* infection, the diagnosis, treatment, and progression of the condition can be more complex [1–3]. The urea breath test (UBT) can detect *H. pylori*, but might yield false-positive results in the presence of autoimmune gastritis due to altered gastric conditions [4]. AIG can alter the gastric environment, leading to false-positive UBT results due to the overgrowth of urease-producing bacteria other than *H. pylori* [4]. Also, both autoimmune gastritis and *H. pylori* infection independently increased the risk of gastric cancer. In AIG, the body’s immune system attacks the stomach lining, leading to chronic inflammation and potential developmental of gastric cancer [4, 5]. On the other hand, *H. pylori* infection causes chronic gastritis, which can also progress to gastric cancer over time [4]. Therefore, the combined presence necessitates vigilant monitoring and possibly more aggressive management. Even after *H. pylori* eradication, patients with autoimmune gastritis require ongoing management to address nutrient deficiencies and to monitor for complications.

Even though we now know more about the phenotypic alterations or cascades that occur in AIG, there is still confusion surrounding the secondary preventive efforts, histological reporting practices, and etiopathogenic pathways underlying the disease. Here, we report the case of an elderly woman with AIG after *H. pylori* eradication.

## Case report

**Figure 1:**
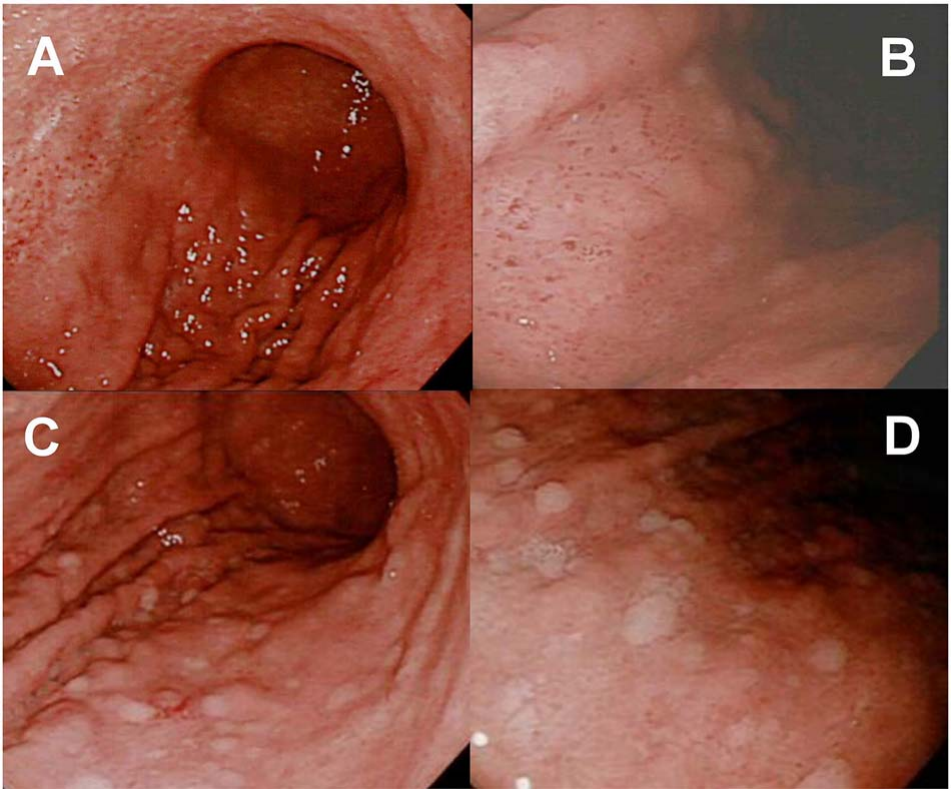
(A & B) endoscopic findings before *H. Pylori* eradication: Diffuse redness, mucosal swelling, and enlarged folds are observed in the gastric corpus. (C&D) endoscopic findings after eradication: Remnants of the oxyntic mucosa sticky adherent dense mucus and scattered minute whitish protrusions were identified at the greater curvature site of the gastric corpus.

**Figure 2:**
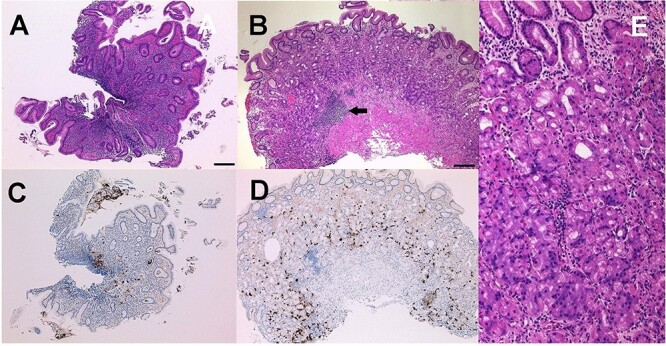
(A) Biopsy specimens from the greater curvature of the gastric corpus before *H. Pylori* eradication; moderately infiltrating inflammatory neutrophilic cells in the gastric superficial mucosa are shown. (bar = 200 μm). (B) Biopsy specimens from the greater curvature of the gastric corpus after *H. Pylori* eradication; infiltrating lymphocytes increased near the muscularis mucosa. (bar = 200 μm). The arrow shows infiltrating lymphocyte sites. (C & D) biopsy specimens from the greater curvature of the gastric corpus before (C) and after (D) *H. Pylori* eradication. Chromogranin A-positive cells increased in the superficial mucosa after (D) *H. Pylori* eradication. (E) Biopsy specimens from the greater curvature of the gastric corpus after *H. Pylori* eradication; infiltrating inflammatory neutrophilic cells decreased in the superficial mucosa but infiltrating lymphocytes increased in the sub-superficial mucosa.

A 70-year-old female patient presented with gastric pain. In November 2021, esophagogastroduodenoscopy (EGD) was performed, which revealed diffuse redness, mucosal swelling, enlarged folds in the gastric corpus (Fig. 1A and B), and chronic atrophic gastritis (open type: O-1). The endoscopic findings were matched to predict *H. pylori i*nfection status [1]. Biopsy specimens from the greater curvature of the gastric corpus revealed moderately infiltrating inflammatory neutrophilic cells in the superficial gastric mucosa (Fig. 2A). Consistent with this, it has been reported that surface epithelial injury, neutrophilic infiltration, and chronic inflammatory infiltration are all closely related to *H. pylori* infection [2]. Her serum anti-*H. pylori* IgG antibody level was high (65 U/ml)(0–3), and *H. pylori* was detected in the gastric biopsy specimen of the patient’s gastric corpus. The patient was treated with eradication therapy (VONOSAP® Pack 400). In June 2023, the patient again experienced gastric pain. Stool antigen test results for *H. pylori* were negative. The patient also underwent a 13C-urea breath test, but the result was positive. In the context of autoimmune gastritis, the 13C-urea breath test can sometimes yield false-positive results. This is because autoimmune gastritis can lead to changes in the stomach lining that might affect the test’s accuracy, due to the altered gastric environment and bacterial overgrowth [1]. Furthermore, in June and October 2023, she gained the same results of EGD, which revealed remnants of the oxyntic mucosa sticky adherent

dense mucus and scattered minute whitish protrusions at the greater curvature site of the gastric corpus (Fig. 1 C and D). These endoscopic findings were matched to predict AIG [3]. Biopsy specimens from the greater curvature site of the gastric corpus revealed that infiltrating lymphocytes increased near the muscularis mucosa, although these lymphocytes did not directly indicate inflammatory cells (Fig. 2B), and that infiltrating inflammatory neutrophilic cells decreased in the superficial mucosa, Chromogranin A-positive cells increased (Fig. 2C and D) in the superficial mucosa (Fig. 2C and D), and infiltrating lymphocytes increased in the superficial mucosa (Fig. 2E). Consistent with this, it was reported that the early phase of AIG is characterized by diffuse basal-predominant inflammation within the mucosa [5].

In July 2023, Laboratory investigations revealed 15.1 g/dl serum hemoglobin (11.6–14.8). Her blood ferritin and vitamin B12 were 149.4 ng/mL (5.0–179.0) and 640 pg/mL (233–914). In December 2023, these revealed 14.9 g/dl serum hemoglobin. Her blood vitamin B12, gastrin, and parietal cell antibody levels were 690 pg/mL, 105 pmol/L (11.9–46.9), and 1:10 (<10), respectively. At this time, she had no spinal cord nerve disease or complications of other autoimmune diseases.

## Discussion

Some studies have reported that *H. pylori* eradication increases AIG [6]. In contrast, others reported that the eradication did not increase AIG [7]. There can be much greater comparable instances wherein AIG stays inconspicuous beneathneath *H. pylori* energetic gastritis and is hastily exacerbated after *H. pylori* eradication therapy, consisting of this provided case. To remedy this problem, we wanted to take note of peculiar AIG instances hidden with the aid of *H. pylori* gastritis. However, it is difficult to hit upon and diagnose pre-atrophic AIG during ongoing *H. pylori* gastritis.

It has been reported that in AIG, the destruction of parietal cells in the stomach can lead to elevated levels of Chromogranin A [8]. In the present case, after eradication, infiltrating inflammatory neutrophilic cells decreased in the superficial mucosa, Chromogranin A-positive cells increased in the superficial mucosa, and infiltrating lymphocytes increased in the sub-superficial mucosa. In addition, this patient tested positive for parietal cell antibodies. Furthermore, hypergastrinemia or an increased amount of gastrin in the blood is one of the main characteristics of AIG. This happens as a result of decreased stomach acid production brought on by parietal cell destruction, which causes the body to create more gastrin in an effort to increase acid production [8].

Before *H. pylori* eradication, the patient had atrophic gastritis (open type: O-1), and there was no difference between before and approximately 2 years after eradication. Even after *H. pylori* is successfully eradicated, atrophic alterations in the stomach mucosa, especially in the corpus, may worsen in one instance of AIG over several years [9]. Conversely, there has also been a report of improvement in serological markers and histological results following *H. pylori* eradication, along with regression of gastric mucosal atrophy in an AIG patient after 2.5 years [10].

In conclusion, this case suggests that an elderly woman developed AIG after *H. pylori* eradication. AIG is characterized by chronic inflammation of the stomach lining due to the immune system attacking parietal cells. Long-term proton pump inhibitor use can lead to changes in the stomach lining, which may complicate autoimmune gastritis [4]. She had been taking a proton pump inhibitor for gastric pain for several weeks, which was previously prescribed by another physician. Because the period is very short, we speculate that the possibility that this drug causes AIG is low. Because prospective studies on the natural history of AIG are lacking, further studies regarding the detailed time course are required.

## CRediT statement

TK performed the endoscopic procedures, drafted the manuscript, and managed the patient. SN performed pathological diagnosis. All authors read and approved the final report.
